# No association between peripheral serotonin-gene-related DNA methylation and brain serotonin neurotransmission in the healthy and depressed state

**DOI:** 10.1186/s13148-024-01678-y

**Published:** 2024-05-27

**Authors:** S. E. P. Bruzzone, B. Ozenne, P. M. Fisher, G. Ortega, P. S. Jensen, V. H. Dam, C. Svarer, G. M. Knudsen, K. P. Lesch, V. G. Frokjaer

**Affiliations:** 1 Neurobiology Research Unit, Copenhagen University Hospital Rigshospitalet, Copenhagen, Denmark; 2 Department of Clinical Medicine, Faculty of Health and Medical Sciences, University of Copenhagen, Copenhagen, Denmark; 3 Department of Public Health, Section of Biostatistics, University of Copenhagen, Copenhagen, Denmark; 4 Department of Drug Design and Pharmacology, University of Copenhagen, Copenhagen, Denmark; 5 Division of Molecular Psychiatry, Center of Mental Health, University Hospital Würzburg, Würzburg, Germany; 6 Department of Psychiatry and Neuropsychology, School for Mental Health and Neuroscience (MHeNs), Maastricht University, 6229 ER Maastricht, The Netherlands; 7 Psychiatric Centre Copenhagen, Mental Health Services, Frederiksberg, Capital Region of Denmark Denmark

**Keywords:** Serotonin transporter, 5-HT, Tryptophan hydroxylase 2, TPH2, Serotonin 4 receptor, Depression, Human brain imaging, PET, Mood disorders, Epigenetics, Early life stress

## Abstract

**Background:**

Methylation of serotonin-related genes has been proposed as a plausible gene-by-environment link which may mediate environmental stress, depressive and anxiety symptoms. DNA methylation is often measured in blood cells, but little is known about the association between this peripheral epigenetic modification and brain serotonergic architecture. Here, we evaluated the association between whole-blood-derived methylation of four CpG sites in the serotonin transporter (*SLC6A4*) and six CpG sites of the tryptophan hydroxylase 2 (*TPH2*) gene and in-vivo brain levels of serotonin transporter (5-HTT) and serotonin 4 receptor (5-HT_4_) in a cohort of healthy individuals (*N* = 254) and, for 5-HT_4,_ in a cohort of unmedicated patients with depression (*N* = 90). To do so, we quantified *SLC6A4*/*TPH2* methylation using bisulfite pyrosequencing and estimated brain 5-HT_4_ and 5-HTT levels using positron emission tomography. In addition, we explored the association between *SLC6A4* and *TPH2* methylation and measures of early life and recent stress, depressive and anxiety symptoms on 297 healthy individuals.

**Results:**

We found no statistically significant association between peripheral DNA methylation and brain markers of serotonergic neurotransmission in patients with depression or in healthy individuals. In addition, although *SLC6A4* CpG2 (chr17:30,236,083) methylation was marginally associated with the parental bonding inventory overprotection score in the healthy cohort, statistical significance did not remain after accounting for blood cell heterogeneity.

**Conclusions:**

We suggest that findings on peripheral DNA methylation in the context of brain serotonin-related features should be interpreted with caution. More studies are needed to rule out a role of *SLC6A4* and *TPH2* methylation as biomarkers for environmental stress, depressive or anxiety symptoms.

**Supplementary Information:**

The online version contains supplementary material available at 10.1186/s13148-024-01678-y.

## Background

Most psychiatric disorders, including major depressive disorder (MDD), arise from a complex etiology, with contributions from genetic and environmental factors. The serotonin system mediates a variety of different functions from the very early stages of development and throughout life, including cognition, mood and sleep as well as adaptation to environmental challenges [1–4]. For instance, serotonin-mediated neuroplasticity has been suggested to allow us to adapt to the ever-changing environment [5]. In this case, alterations in serotonin function might translate into resilience or vulnerability to MDD [2, 6].

DNA methylation of genes coding for key regulators of the serotonin system, such as the serotonin transporter (*SLC6A4*) and the tryptophan hydroxylase 2 gene (*TPH2*), has been proposed as a possible gene-by-environment mechanism involved in several psychiatric disorders, including MDD [7, 8]. However, DNA methylation is often measured in peripheral samples (e.g. blood or saliva) and little is known of the effect of this modification on in-vivo brain serotonin transmission. In this study, we investigated the association between methylation of peripheral *SLC6A4* and *TPH2* and brain proxies of serotonin transmission measured with in-vivo positron emission tomography (PET) imaging.

The serotonin transporter (5-HTT) and tryptophan hydroxylase 2 (TPH2) critically shape serotonin signalling by regulating serotonin levels. Specifically, 5-HTT regulates synaptic levels of serotonin available for neurotransmission and is the main target of selective serotonin reuptake inhibitors (SSRI), the most widely used class of antidepressant medications. TPH2 is the rate-limiting enzyme for serotonin synthesis in the brain [9], thereby directly affecting presynaptic serotonin levels.

A role of *SLC6A4* in gene-by-environment interaction was initially described by Caspi and colleagues [10], reporting that 5-HTTLPR s-carriers, who had lower *SLC6A4* expression, were more vulnerable to stress in terms of developing depressive episodes when experiencing stressful life events. Nonetheless, these findings have not been replicated by all larger studies [11, 12], suggesting that 5-HTTLPR per se may not be as relevant to MDD or anxiety-related traits as previously thought. Instead, a combination of genetic and epigenetic factors may affect *SLC6A4* gene expression levels [13] in a way that may be relevant to the development of psychopathology [14].

Over the last two decades, several studies have pointed to a possible role of DNA methylation levels in the transcriptional control region of *SLC6A4* as a marker of gene-by-environment interaction [7, 15–17]. Specifically, alterations in *SLC6A4* methylation have been associated with recent [15] and early life stress [7, 18], depressive symptoms [19], panic disorder [16] and likelihood to respond positively to antidepressant treatment [20, 21], although the relation with depressive symptoms and antidepressant treatment outcome was not confirmed by all studies [20, 22].

More recently, methylation of *TPH2* gene has also been suggested as a biomarker for vulnerability to depression and antidepressant treatment outcome [23, 24].

DNA methylation at cytosine-guanine dinucleotides (CpG) is a common epigenetic modification which can affect gene expression in response to environmental cues [25]. Early studies reported an association between *SLC6A4* methylation and 5-HTT mRNA levels measured in lymphocytes [13] and peripheral whole blood [26, 27]. However, it is not known whether such altered peripheral 5-HTT mRNA levels also correlate with brain 5-HTT protein levels. Given the fundamental role of 5-HTT and TPH2 in serotonin neurotransmission, understanding whether peripheral *SLC6A4* or *TPH2* methylation mirrors serotonin brain architecture is essential to interpret previous findings and to shed light on the role of peripheral methylation in the context of health and disease, e.g. psychiatric disorders.

Indeed, it is important to note that DNA methylation is cell-type specific [28]. However, as brain tissue of living human participants is mostly unavailable for biomarker assessment, blood and saliva are the most used tissue types for the investigation of DNA methylation. Peripheral blood and postmortem brain DNA methylation partially correlate at multiple CpG sites, but there is not a perfect correspondence between the two tissues [29–31]. Evaluating DNA methylation associations with in vivo brain serotonin markers allows to estimate its relevance as a peripheral marker of serotonin neurotransmission. However, to our knowledge, no study has investigated whether peripheral methylation of *SLC6A4* or *TPH2* is associated with brain levels of 5-HTT or with other markers of serotonin neurotransmission, such as serotonin 4 receptor (5-HT_4_), a post-synaptic serotonin receptor that has been proposed as a biomarker for brain serotonin tonus [32]. Only one study reported an association between *SLC6A4* promoter methylation and brain serotonin synthesis measured in terms of brain tryptophan levels [27].

PET imaging allows quantification of serotonin system protein levels in the living brain [33, 34]. In this study, we used PET scans of 254 healthy participants and 90 patients with MDD to determine the relation between peripheral *SLC6A4* and *TPH2* methylation and two key features of the serotonergic brain signalling system, i.e. 5-HTT and 5-HT_4_, imaged with combined with [^11^C]DASB and [^11^C]SB207145 PET radiotracers, respectively. Both the 5-HTT and 5-HT_4_ are known to play a role in healthy brain function and in MDD pathology and can be considered as key markers for serotonin neurotransmission [35–38].

Furthermore, as primary sensitivity analyses, we evaluated the association between DNA methylation and self-reported early life stress and stressful life events, as well as state measures of perceived stress and anxiety and depressive symptoms in 297 healthy participants.

Finally, blood is a heterogeneous tissue containing different cell types. Interindividual differences in blood cell proportions can be a source of bias on DNA methylation measurements carried out on whole blood [39], hindering comparability between individuals. Nonetheless, while epigenome-wide studies routinely correct for blood cell proportions [40], most of previous studies linking *SLC6A4* and *TPH2* methylation to environmental stress [15, 41] or psychiatric conditions [7, 16, 21, 23] did not account for blood cells proportions. Thus, we used blood cell counts to estimate blood cell proportions in a subgroup of participants for whom this information was available. Then, we included cell proportions in all our statistical models as secondary sensitivity analyses.

## Methods

### Participants

**Fig. 1:**
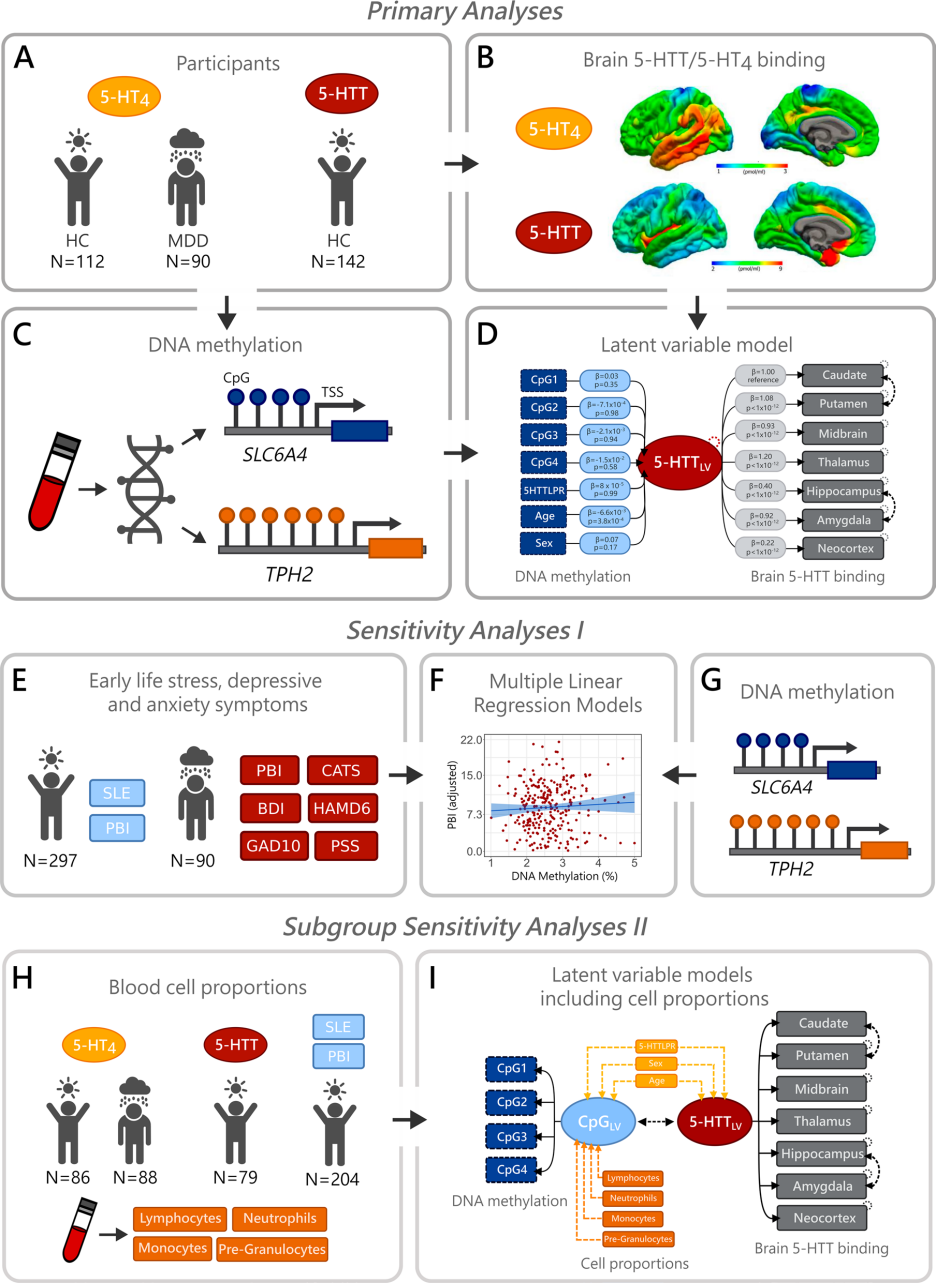
Overview of the data and methods used in this study. **a**, **b**, **c**, **d** depict the primary analyses, in which a latent variable model was used to determine the association between peripheral *TPH2* and *SLC6A4* methylation and brain levels of 5-HTT and 5-HT_4_. **e**, **f**, **g** describe the sensitivity analyses evaluating the association between *SLC6A4*/*TPH2* methylation and measures of environmental stress, depressive and anxiety symptoms. **h** and **i** show sensitivity analyses used to evaluate potential influence of blood cell proportions in the A-G analyses. Abbreviations: 5-HT_4_: serotonin 4 receptor; 5-HTT: serotonin transporter; CpG: CpG site; TSS: transcription start site; SLE: stressful life events; PBI: parental bonding inventory; BDI: Beck’s depressive index; GAD10: generalized anxiety disorder 10-item; CATS: childhood abuse trauma scale; HAMD6: Hamilton depressive rating scale 6; PSS: perceived stress scale; CpG_LV_: latent variable including all CpG methylation values

All participants included in this study were recruited as part of neuroimaging projects conducted at Neurobiology Research Unit, Copenhagen University Hospital Rigshospitalet, in compliance with the Declaration of Helsinki and Good Clinical Practice guidelines. An overview of the methods is depicted in Fig. 1.

#### Healthy Cohort

We included data of healthy volunteers from the Cimbi database and biobank [42]. The data was included based on the following criteria: (1) availability of [^11^C]DASB PET or [^11^C]SB207145 PET; (2) availability of whole blood or buffy coat samples matching scan date (blood samples drawn maximum one week before or after the PET scan were also included for *N* = 3) and (3) self-identification with European ancestry. Before inclusion in any of the original studies, all participants were screened for psychiatric disorders and underwent a physical and neurological examination. Participants with a history of psychiatric illness or current use of psychotropic drugs or drugs potentially affecting PET measurements were excluded.

We identified a cohort of 142 participants with [^11^C]DASB PET scans and a cohort of 112 participants with [^11^C]SB207145 PET scans. Demographic data relative to the cohorts included in the study are depicted in Table 1.

**Table 1:** Demographics of the participants included in the primary analyses

		5-HTT	5-HT_4_	
		HC	HC	MDD
*N*		142	112	90
Age (mean ± s.d. [min–max])		29.0 ± 11.8 [18.4–80.3]	29.3 ± 12.9 [19.2–86.2]	26.7 ± 7.6 [18.2–56.4]
Sex (F/M)		81/61	65/47	64/26
PET scanner (A/H)		50/92	20/92	90/-
MRI scanner		*T* = 92/*V* = 50	*T* = 59/*P* = 50/*V* = 3	*P* = 90
[^11^C]DASB/SB207145-injected mass (μg)		0.045 ± 0.041	0.023 ± 0.024	0.013 ± 0.015
[^11^C]DASB/SB207145-injected dose (MBq)		546 ± 83.7	557 ± 91.3	578 ± 56.3
Cerebellum AUC (Bq ml^−1^)		18,200 ± 3450	10,200 ± 2450	10,200 ± 2520
*SLC6A4* methylation (%)	CpG1	2.65 ± 0.67 [1.4–5.05]	2.54 ± 0.58 [1.24–4.71]	2.3 ± 0.55 [1.28–3.94]
	CpG2	3.61 ± 0.80 [1.66–5.7]	3.53 ± 0.77 [1.75–5.42]	3.29 ± 0.71 [1.88–5.26]
	CpG3	2.97 ± 0.73 [1.56–5.72]	2.92 ± 0.74 [1.56–5.9]	2.73 ± 0.61 [1.5–4.38]
	CpG4	3.94 ± 1.07 [2.44–11.8]	3.78 ± 0.85 [1.4–8.48]	3.54 ± 0.76 [2.06–5.8]
*TPH2* methylation (%)	CpG1	3.22 ± 0.66 [1.89–5.88]	3.26 ± 0.65 [2–5.58]	2.7 ± 0.49 [1.47–4.18]
	CpG2	3.14 ± 0.67 [1.8–4.51]	3.14 ± 0.69 [1.96–5.38]	2.88 ± 0.53 [1.65–3.98]
	CpG3	2.91 ± 0.62 [1.7–4.68]	2.93 ± 0.75 [1.74–8.18]	2.6 ± 0.54 [1.54–4]
	CpG4	2.27 ± 0.51 [1.27–3.93]	2.33 ± 0.61 [1.28–5.96]	2.02 ± 0.49 [1.14–3.66]
	CpG5	3.25 ± 0.73 [1.92–7.18]	3.43 ± 0.88 [1.87–8.02]	3.06 ± 0.66 [1.94–5.26]
	CpG6	3.34 ± 0.80 [1.72–8.72]	3.42 ± 1.05 [1.7–10.8]	2.96 ± 0.52 [1.96–4.78]
*SLC6A4* 5-HTTLPR/rs25531 (L_A_L_A_ / S-)		41/101	36/76	24/62
*TPH2* rs4570625 (GG/TX)		90/52	71/40	54/34
*BDNF* Val/Met (rs6265) (Val/Val / Met-carriers)		87/50	60/52	46/20
*MAOA* rs1137070 (CC/T-)		75/58	–	–
Blood cells counts available (yes/no)		79/63	86/26	88/2

*HC:*Healthy Control*; MDD:* patients with major depressive disorder*; 5-HTT:* serotonin transporter*; 5-HT*_*4*_*:* serotonin 4 receptor*; F:* female; *M:* male; *PET*: positron emission tomography; *A*: GE-Advance PET scanner; *H*: HRRT PET scanner; MRI: magnetic resonance imaging; *T*: Trio MRI scanner; *V*: Verio MRI scanner; *P*: Prisma MR scanner; *μg*: microgram; *MBq*: megabecquerel; *Bq ml*^*−1*^: becquerel per milliliter; *AUC*: area under the curve (i.e., cerebellum reference region time activity curve); *SLC6A4*: serotonin transporter gene; *TPH2*: tryptophan hydroxylase 2 gene; 5-HTTLPR, serotonin-transporter-linked promoter region; *BDNF*: brain-derived neurotrophic factor; *MAOA*: monoamine oxidase A gene

To evaluate the potential association between DNA methylation and early life stress history or state measures of perceived stress, depressive or anxiety symptoms, data from an additional 43 healthy participants without PET scans was available, resulting in a total of 297 healthy participants. Demographics for the participants included in all analyses are depicted in Table S1.

#### MDD patient cohort

We included baseline data from 90 unmedicated patients with moderate to severe unipolar MDD that were originally part of the NeuroPharm-1 study [43], an open-label, non-randomized longitudinal clinical trial. Patients were included based on the availability of both blood samples and [^11^C]SB207145 PET scans that were collected no more than one week apart.

The primary outcome of the trial involved measures of molecular neuroimaging and cognitive functions and is described in previous publications [36, 43, 44]. Shortly, previously unmedicated patients with MDD were recruited for an open-label clinical trial aiming to uncover biomarkers predicting clinical outcome after 12 weeks of antidepressant treatment. In this study, we included blood samples, PET scans and psychometric data collected at baseline. Analyses carried out in this study involving *SLC6A4* are planned secondary analyses. Analysis including *TPH2* are unplanned exploratory analyses. Participants were evaluated in face-to-face interviews and diagnosed by a certified psychiatrist. Individuals between 18–65 years of age, scoring > 17 in the Hamilton Depression Rating Scale 17 items (HAMD_17_)[45] and who were unmedicated for at least two months before the start of the trial were included in the study. Additional details about the trial as well as inclusion and exclusion criteria are specified in [43].

### DNA methylation analysis

*SLC6A4* methylation percentages were estimated at four CpG sites (Table S2) that were previously linked to clinical phenotypes, including depressive symptoms [7, 16, 26, 46], early-life adversities [7], recent environmental stress [15], antidepressant treatment outcome [20, 21] and panic disorder [16]. *TPH2* methylation was estimated at 6 CpG sites (Table S2) based on previous studies showing an association with gene expression (13, 26, 28), early life stress, depressive symptoms, antidepressant treatment outcome [23, 24, 47] and attention deficit hyperactivity disorder [48].

Genomic DNA was isolated from peripheral blood cells from whole blood or buffy coat samples that were stored at − 20 °C (MDD patient cohort) or − 80 °C (healthy cohort) in EDTA tubes. DNA was purified using the FlexiGene Kit (Qiagen, Hilden, Germany), according to the manufacturer’s protocol. 500 ng DNA of each sample were bisulfite-converted using the EpiTect 96 Bisulfite Kit (Qiagen). The sequence of interest was amplified via polymerase chain reaction (PCR) using the PyroMark PCR Kit (Qiagen) and a forward (F) and a reverse (R) biotinylated primer (**Table S3**). The quality of PCR amplification was visually evaluated using gel electrophoresis. The target DNA sequence was isolated and then sequenced using the PyroMark Q96 ID (Qiagen) pyrosequencing system, with target-specific primers (**Table S3**). CpG methylation rates (in %), pyrograms and quality reports were obtained using the PyroMark software (Qiagen). Analyses were run in duplicates and pairs of duplicates differing more than 3% from each other were excluded from the analyses. Average DNA methylation value between each pair of duplicates was used for statistical analyses. Pyrograms and quality reports provided by PyroMark were used to quality check the data. Commercially available (Epitect PCR Control DNA Set, Qiagen) fully methylated, fully non-methylated and 50%-methylated DNA samples as well as DNase free H_2_O were included in all experiments as controls. Methylation data of two samples for *SLC6A4* and three samples for *TPH2* were excluded due to failed bisulfite conversion, as indicated by the PyroMark software.

### Genotyping

All samples were genotyped for *SLC6A4* 5-HTTLPR and rs25531, *TPH2* rs4570625, *BDNF* rs6265 and *MAOA* rs1137070 polymorphisms. Genotyping for *SLC6A4* 5-HTTLPR and rs25531, *BDNF* rs6265 and *MAOA* rs1137070 was performed as previously described [35, 49–51]. Hardy–Weinberg equilibrium was tested using Chi-squared test in R. Table 1 and **S1** show allele frequencies within all cohorts.

### PET and MR data acquisition and processing

The acquisition, preprocessing and quantification of [^11^C]SB207145 and [^11^C]DASB PET and MR images has been previously reported [37, 50, 52]. For each participant, both PET and concomitant MR scans were acquired. MR scans coregistered to PET were used to delineate brain regions and quantify regional PET signal.

Shortly, all participants were scanned for a 120-min ([^11^C]SB207145) or a 90-min ([^11^C]DASB) dynamic scan after bolus injection of the respective radioligand. Two different PET scanners were used for data collection: a High-resolution Research Tomography (HRRT) PET scanner (CTI/Siemens) with an approximate in-plane resolution of 2 mm, or an 18-ring GE-Advance PET scanner (General Electric, Milwaukee, USA) with an approximate in-plane resolution of 6 mm. T1-weighted MPRage images were acquired using three different Siemens 3-Tesla magnetic resonance (MR) scanners: Prisma, Trio or Verio. Regions of interest (ROI) were automatically delineated using PVElab and the individual T1-weigthed images [53]. Mean time-activity curves for average grey matter voxels in each hemisphere was determined using the Simplified Reference Tissue Model for [^11^C]SB207145 scans and multilinear reference tissue model (MRTM/MRTM2) for [^11^C]DASB scans. Cerebellum (except for vermis) was used as a reference region for all scans. The non-displaceable binding potential (binding or BP_ND_) was used as an outcome measure of tracer binding (and therefore as an estimate of 5-HTT and 5-HT_4_ levels) for both tracers.

### Statistical analyses

All statistical analyses were conducted in R v4.1.2 [54].

#### Primary analyses

The association between *SLC6A4*/*TPH2* methylation and 5-HTT or 5-HT_4_ was evaluated using three different linear latent variable models (LVM): one for healthy controls with DASB scans (5-HTT binding), one for healthy controls with SB scans (5-HT_4_ binding) and one for MDD patients with SB scans. LVM is a type of multivariate linear regression that allows to model associations between a variable of interest and the shared variance of a set of inter-correlated variables (e.g. 5-HTT or 5-HT_4_ binding in different brain regions).

Regions of interest (ROI) for the 5-HTT LVM were chosen based on 5-HTT distribution in the human brain [55] and comprised caudate, amygdala, hippocampus, putamen, thalamus, midbrain and neocortex. Similarly, ROIs for the 5-HT_4_ LVMs include caudate, putamen, hippocampus and neocortex, reflecting brain regions across low, moderate to high density of 5-HT_4_ receptor in these areas [55] and aligning with previous findings investigating the 5HT_4_ receptor system and MDD [32, 56].

Analyses were carried out in R and the LVMs were modelled using the *lava* v 1.6.10 [57] package. First, the shared correlations of regional 5-HT_4_ or 5-HTT binding were modelled into a latent variable for each model (referred to as 5-HT_4LV_ or 5-HTT_LV_ respectively). Next, *SLC6A4* CpG1-CpG4 or *TPH2* CpG1-6 methylation and the covariate effects were modelled on the 5-HTT_LV_. In all models, covariates included age, sex, PET scanner type (Advance vs HRRT) and MR scanner type (Prisma vs Trio vs Verio). 5-HTTLPR/rs25531 genotype was included in the statistical models including *SLC6A4* methylation based on previous studies suggesting a combined effect of genotype and DNA methylation on 5-HTT transcription [13, 17]. Similarly, *TPH2* rs4570625 was included in the models evaluating *TPH2* methylation [48]. Models evaluating associations between DNA methylation and 5-HT_4_ binding also included information of 5-HTTLPR/rs25531 and *BDNF* rs6265, based on previous findings [58]. In addition, *BDNF* rs6265 and *MAOA* rs1137070 genotype information, which have been previously shown to affect 5-HTT [49, 50, 58], were included in a separate model as sensitivity analyses, as information for the latter genotype was not available for all subjects. Region-specific effects of each CpG site were evaluated as the product of the CpG effect on the latent variable multiplied by the loading of each region on the latent variable and were used as a measure of effect sizes.

PET and MR scanner type were modelled as region-specific effects, based on previous findings [58]. Additional covariance links were identified using an iterative procedure where score tests are used to detect model misspecification. *P*-values for these score test were adjusted using Benjamini–Hochberg [57].

**Fig. 2:**
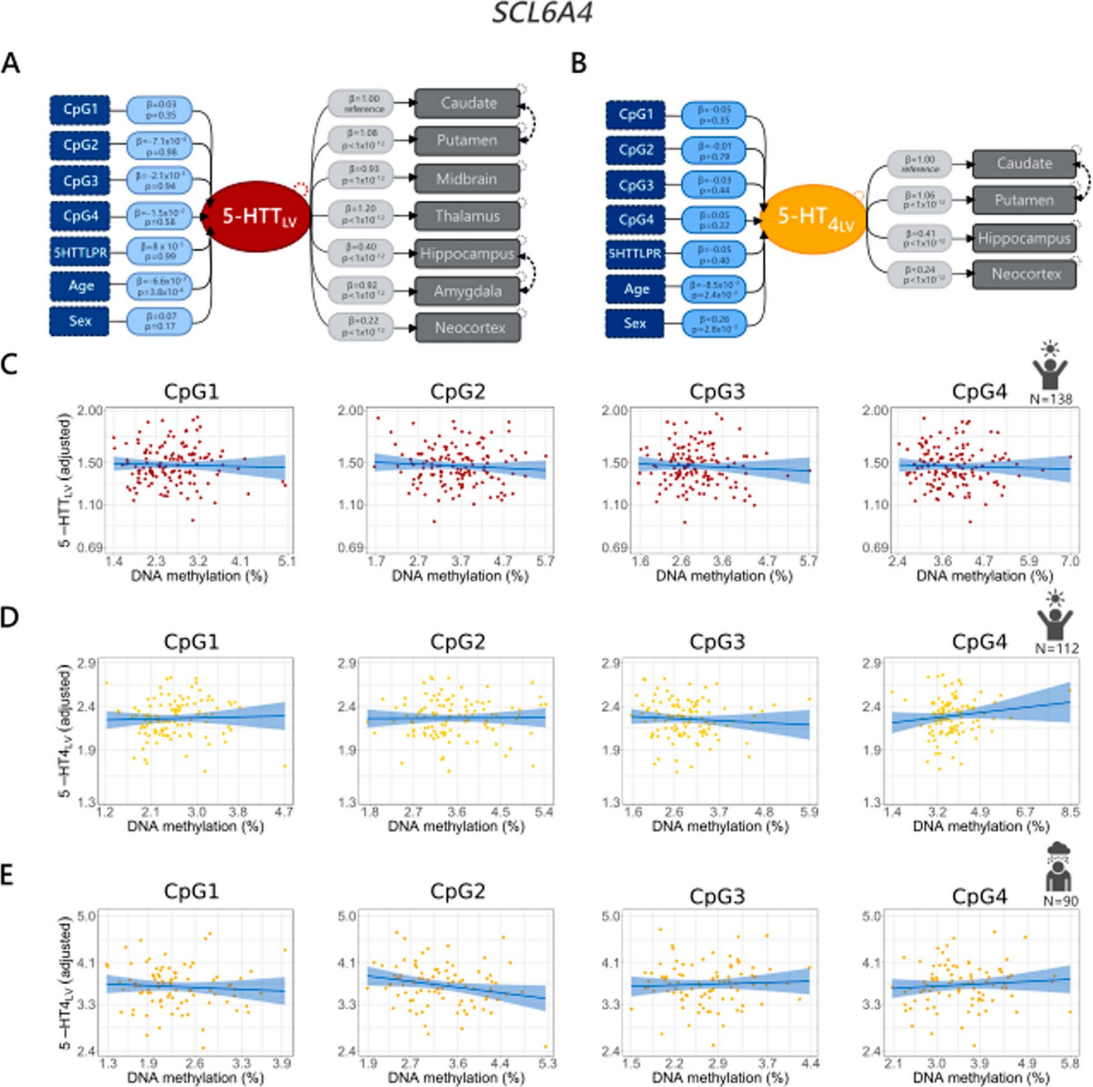
Associations between peripheral *SLC6A4* methylation and brain 5-HTT binding (**a**) or 5-HT_4_ binding (**b**). Blue dashed boxes depict the *SLC6A4* CpG sites and the covariates included in the model. The light blue boxes indicate the CpG and covariate effects on the latent variable (5-HTT_LV_ or 5-HT_4__LV_). Dark grey boxes to the right represent the observed 5-HTT or 5-HT_4_ binding in the brain regions of interest. *β* values refer to the parameter estimates; they are reported either with their respective *p*-values or with their 95% confidence intervals. Dashed arrows connecting brain regions show interregional correlations, while dashed circles on the brain regions show error estimates. For representation purposes, PET and MR scanner covariates are not reported in the **a** and **b** models. Similarly, although included in the 5-HT_4_ latent variable model, 5-HTTLPR/rs25531 and *BDNF* rs6265 genotypes are not reported in (**b**). Scatter plots in **c** and **d** depict the relation between *SLC6A4* methylation and 5-HTT_LV_ or 5-HT_4LV_ in healthy controls (**c**, **d**), while the relation between *SLC6A4* methylation and 5-HT_4_ binding in patients with MDD is shown in (**e**)

**Fig. 3:**
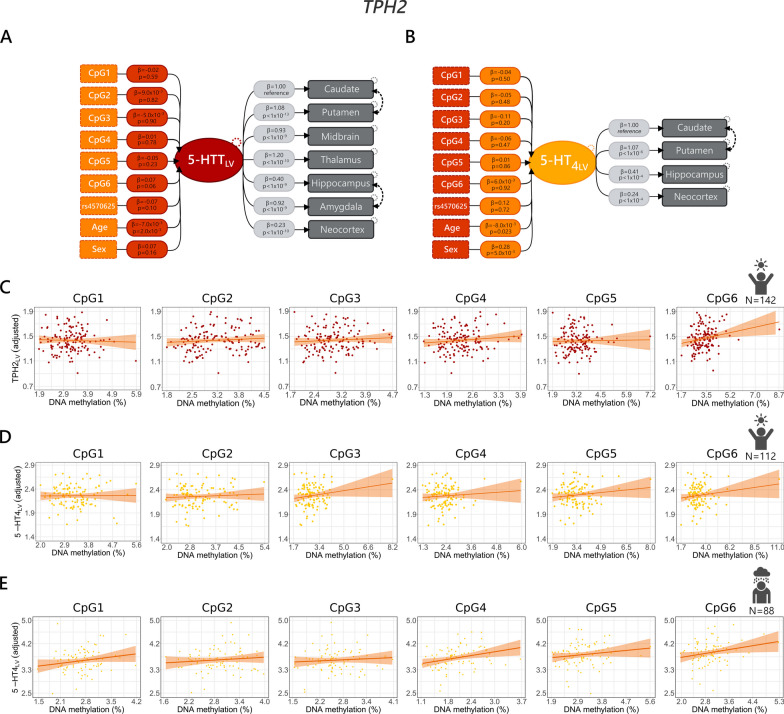
Associations between peripheral *TPH2* DNA methylation and brain 5-HTT binding **a** or 5-HT_4_ binding **b** in the healthy cohort. Orange dashed boxes to the left depict the *TPH2* CpG sites and the covariates included in the model. Rs45706210 stands for *TPH2* rs45706210 G/T SNP. For representation purposes, PET and MR scanner covariates are not reported in the **a** and **b** models. Similarly, although included in the 5-HT_4_ latent variable model, 5-HTTLPR/rs25531 and *BDNF* rs6265 genotypes are not reported in (**b**). Scatter plots in **c** and **d** depict the relation between *TPH2* methylation and 5-HTT_LV_ or 5-HT_4LV_ in healthy controls (**c**, **d**), while the relation between *TPH2* methylation and 5-HT_4_ binding in patients with MDD is showed in (**e**)

The LVMs used for primary analyses are graphically represented in Figs. 2 and 3.

Caudate was used as a reference region in all LVMs. Thus, covariate effects can be interpreted as effects on caudate binding. Statistical significance was set at *p* < 0.05 for all the statistical models.

#### Sensitivity analyses I: DNA methylation vs measures of environmental stress, depressive and anxiety symptoms

Multiple linear regression models were used to explore the relation between methylation of each CpG site and measures of environmental stress in both healthy participants and MDD patients. Associations with depressive and anxiety symptoms were also evaluated in the MDD patients. Data of all the healthy controls were pooled together with data of 58 additional healthy participants, for a total of *N* = 297 (**Table S1**). Data from the MDD patients were the same as those used for the LVM analyses. The stressful life events (SLE) questionnaire was used as an estimate of both lifetime (total SLE score) and recent stress (recent SLE score) in the healthy cohort. The parental bonding inventory (PBI) was used as a proxy estimate of early life stress in both the healthy and the MDD cohorts. Scores from both parents were combined into a measure for the “care” (PBI care score) and one for the “overprotection” (PBI overprotection score) subscales. In addition, models exploring the association between *SLC6A4* or *TPH2* DNA methylation and the following measurements were carried out in the MDD cohort: (1) Beck’s Depression Inventory (BDI) indexing recent depressive symptoms; (2) childhood abuse trauma scale (CATS) as a measure of early life stress; (3) generalized anxiety disorder 10-item (GAD10); (4) Hamilton depression rating scale 6 item (HAMD_6_) indexing current depressive symptoms; (5) perceived stress scale (PSS) indexing recent stress symptoms.

All statistical models included age, sex and genotype (5-HTTLPR in the case of *SLC6A4* or rs4570625 for *TPH2*) as covariates. Bonferroni correction for four and six tests was applied for analyses including *SLC6A4* and *TPH2* data, respectively (*SLC6A4*: *p* = 0.01; *TPH2*: *p* = 0.008).

#### Sensitivity analyses II: analyses accounting for cell type proportions

Sensitivity analyses were conducted to evaluate whether different blood cell type proportions affected the associations evaluated in the primary analyses and in the primary sensitivity analyses. Blood cell counts information was available only for a subset of the total participants used in the analyses (Table 1; panel H of Fig. 1). Corrections were done for lymphocytes, monocytes, granulocyte precursors and neutrophils proportions. The term granulocyte precursors used here refers to the sum of granulocyte precursors metamyelocytes, myelocytes and promyelocytes. Cell proportions were calculated by dividing the cell counts of each cell type by the number of leukocytes, multiplied by 100.

For the models evaluating the association with brain 5-HTT or 5-HT_4_ levels, the correction for cell type involved first modelling two latent variables, one including the shared correlations among DNA methylation across the four CpG sites (CpG_LV_) and one including the shared correlations of 5-HTT (5-HTT_LV_) or 5-HT_4_ binding (5-HT_4LV_) across model-specific ROIs. CpG_LV_ was adjusted for age, sex, genotype and cell proportions by regressing out all cell proportions but neutrophils proportions (CpG_LV+cells_). Finally, the covariance between CpG_LV+cells_ and 5-HTT_LV_ or 5-HT_4LV_ was estimated (panel I of Fig. 1).

To account for cell type proportions when evaluating the association between peripheral *SLC6A4* or *TPH2* DNA methylation and measures of environmental stress, depressive or anxiety symptoms, LVMs were used instead of linear regressions. For a given gene, a latent variable reflecting DNA methylation at all CpG sites was modelled (CpG_LV_) and adjusted for cell types (CpG_LV+cells_). Next, for every psychometric measurement, LVMs containing CpG_LV+cells_ were regressed out on the psychometric score.

In all models including CpG_LV+cells_, the effect of cell types was tested using a likelihood ratio test between the LVM including CpG_LV_ (without adjustment for cell proportions) and the corresponding LVM including CpG_LV+cells_ (adjusted for cell proportions). Whenever significant, cell type specific effects estimated by the LVM were reported without adjustment for multiple comparisons.

## Results

### Genotyping

Alleles were in Hardy–Weinberg equilibrium (*p* > 0.1) in all cohorts used for statistical analyses (5-HTT, MDD and healthy participants used in sensitivity analyses I) except for rs4570625 in the 5-HT_4_ cohort (*χ*^2^: 6.12; *p* = 0.01). However, rs4570625 did not deviate from Hardy–Weinberg equilibrium for the whole population used in this study (*N* = 389; *χ*^2^: 0.34; *p* = 0.56), suggesting that the lack of equilibrium might be due to chance and not to biases in genotyping.

### Association between peripheral DNA methylation and brain serotonergic markers

Loadings, i.e. parameters evaluating the association between the latent variable and the 5-HTT or 5-HT_4_ binding values, were all significantly different from 0 (all *p* < 10^–4^), indicating evidence for shared variance among the 5-HTT and 5-HT_4_ in the respective ROIs.

The LVMs did not reveal a statistically significant association between *SLC6A4* or *TPH2* methylation and 5-HTT_LV_ or 5-HT_4LV_ in the healthy cohort nor the cohort of MDD patients (unadjusted *p*-values ranged between 0.06 and 0.97; Table 2). A graphical representation of the LVMs including the results is reported in Figs. 2 and 3.

**Table 2:** Results of latent variable models (LVM) evaluating the association between *SLC6A4*/*TPH2* methylation and 5-HTT and 5-HT_4_ brain binding

*SLC6A4*	5-HTT			5-HT_4_					
	HC (*N* = 138)			HC (*N* = 112)			MDD (*N* = 90)		
Variable	*β*	*P*-value	95% CI	*β*	*P*-value	95% CI	*β*	*P*-value	95% CI
CpG1	0.02	0.56	[− 0.051; 0.092]	− 0.01	0.83	[− 0.126; 0.102	− 0.03	0.80	[− 0.229; 0.178]
CpG2	− 0.01	0.78	[− 0.069; 0.052]	− 0.01	0.78	[− 0.106; 0.080]	− 0.13	0.09	[− 0.293; 0.023]
CpG3	− 0.01	0.67	[− 0.084; 0.054]	− 0.07	0.18	[− 0.177; 0.035]	0.11	0.27	[− 0.084; 0.300]
CpG4	− 0.01	0.73	[− 0.073; 0.051]	0.09	0.06	[− 0.003; 0.189]	0.08	0.32	[− 0.076; 0.227]
Age	− 0.01	1.4 × 10^–3^	[− 0.011; − 0.003]	− 0.01	0.01	[− 0.015; − 0.003]	− 0.01	0.18	[− 0.023; − 0.004]
Sex (Male)	0.05	0.32	[− 0.055; 0.164]	0.28	4.2 × 10^–3^	[0.092; 0.460]	0.01	0.95	[− 0.221; 0.235]
5-HTTLPR	− 4.5 × 10^–3^	0.93	[− 0.095; 0.087]	− 0.04	0.57	[− 0.172; 0.097]	0.02	0.87	[− 0.215; 0.252]
*BDNF* rs6265	–	–	–	− 0.08	0.19	[− 0.214; 0.044]	− 1.5 × 10^–3^	0.99	[− 0.211; 0.208]

TPH2	5-HTT			5-HT_**4**_					
	HC (N = 140)			HC (N = 112)			MDD (*N* = 88)		
Variable	*β*	*P*-value	95% CI	*β*	*P*-value	95% CI	*β*	*P*-value	95% CI
CpG1	− 0.02	0.59	[− 0.079; 0.045]	− 0.04	0.50	[− 0.150; 0.075]	0.04	0.76	[− 0.225; 0.305]
CpG2	0.01	0.82	[− 0.066; 0.083]	− 0.05	0.48	[− 0.182; 0.088]	0.02	0.88	[− 0.254; 0.294]
CpG3	− 0.01	0.90	[− 0.095; 0.084]	0.11	0.20	[− 0.059; 0.270]	− 0.25	0.09	[− 0.549; 0.039]
CpG4	0.01	0.78	[− 0.082; 0.109]	− 0.06	0.47	[− 0.243; 0.115]	0.25	0.13	[− 0.076; 0.575]
CpG5	− 0.05	0.23	[− 0.131; 0.032]	0.01	0.86	[− 0.135; 0.162]	0.00	0.97	[− 0.271; 0.262]
CpG6	0.07	0.06	[− 0.004; 0.140]	0.01	0.92	[− 0.120; 0.132]	0.17	0.22	[− 0.110; 0.457]
Age	− 0.01	1.7 × 10^–3^	[− 0.011; − 0.003]	− 0.01	0.02	[− 0.014; − 0.001]	− 0.01	0.10	[− 0.026; − 0.002]
Sex (Male)	0.07	0.16	[− 0.030; 0.176]	0.28	5.0 × 10^–3^	[0.090; 0.472]	0.02	0.86	[− 0.210; 0.250]
*TPH2* rs4570625	− 0.07	0.09	[− 0.150; 0.012]	0.12	0.72	[− 0.558; 0.799]	0.02	0.86	[− 0.204; 0.242]
5-HTTLPR	–	–	–	− 0.03	0.70	[− 0.167; 0.113]	0.09	0.45	[− 0.154; 0.343]
*BDNF* rs6265	–	–	-	− 0.08	0.21	[− 0.212; 0.049]	0.03	0.78	[− 0.193; 0.255]

*HC* healthy controls; *MDD* patients with major depressive disorder; *β* LVM estimated parameters; *95% CI* 95% confidence intervals

In line with previous studies [49, 50, 58, 59] based on the same cohort, we observed: (1) a negative association between age and 5-HTT_LV_ and 5-HT_4LV_ in the healthy cohort (*p* < 0.01) but no association between age and 5-HT_4_ binding in the MDD cohort; (2) a non-significant effect of 5-HTTLPR on 5-HTT_LV_; (3) an association between *MAOA* rs1137070 and 5-HTT_LV_ (*MAOA* T- carriers vs CC, *β*: 0.1, 95% CI: [0.02; 0.18], *p* = 0.01) and between neocortex binding and *BDNF* rs6265 (with lower subcortical binding for met-carriers, estimate (*β)*: − 0.02, 95% CI: [− 0.04; 0.01], *p* = 0.005) in the subset of the sample with this information available (*N* = 130), 4) higher 5-HT_4_ binding in male compared to female participants. Contrary to previous observations based on a subgroup (*N* = 68/112) of the participants included in this study (Fisher et al. 2015: *β*: 0.070, 95% CI: [0.018; 0.122], *p *= 0.008), we did not observe a statistically significant association between 5-HTTLPR or *BDNF* rs6265 genotypes and 5-HT_4LV._ In addition, we did not find any association between *TPH2* rs4570625 and 5-HTT_LV_ or 5-HT_4LV_ (Table 2). Estimated effect sizes and respective 95% CI for effects of each CpG site on each brain region are reported in Tables S4 and S5 for models including *SLC6A4* and *TPH2* respectively. Compared to age, which is known to affect 5-HTT and 5-HT_4_ binding by about 9% and 1% per decade, respectively [59, 60], the effect sizes of our study were minimal. The largest effect sizes in our dataset indicated that 5-HT_4_ binding decreases by 0.14% for each one-unit increase in *SLC6A4* methylation (Table S4) and by 0.24% for each one-unit increase of *TPH2* methylation (Table S5).

### DNA methylation and measures of environmental stress

Results from multiple linear regressions on all cohorts are reported in Table S6 and S7. Among all statistical tests, only three associations reached the threshold for statistical significance before correction for multiple comparisons, and only the association between *SLC6A4* CpG2 and PBI overprotection item remain statistically significant at the 5% level after Bonferroni correction (*β*: − 0.83; *p*_*UNC*_ = 0.01; 95% CI: − 1.48; − 0.19).

### Corrections for cell type

Loadings of DNA methylation at single CpGs onto CpG_LV_ were all significantly different from 0 (all *p* < 0.01). Likewise, loadings of regional 5-HTT or 5-HT_4_ binding significantly loaded onto their corresponding latent variables 5-HTT_LV_ or 5-HT_4LV_ (all *p* < 10^–11^).

Likelihood ratio tests showed an improved model fit when including cell proportions in all the healthy cohorts (all *p* < 0.01). However, in the MDD cohort only the models evaluating the association between *TPH2* methylation and BDI, HAMD_6_, PSS and GAD10 showed improved model fit after adding cell proportions.

Lymphocytes proportion was significantly associated with CpG_LV_ in the *TPH2* models including the healthy cohort (DASB: *β*: 0.017, *p* = 0.017, 95% CI: [0.004; 0.04]; SB: *β*: 0.016, *p* = 0.019, 95%CI: [0.003; 0.03]) and both in the *SLC6A4* and *TPH2* models based on the MDD patients cohort (*SLC6A4*: *β*: 0.014, *p* = 0.03, 95% CI: [0.002; 0.03]; *TPH2*: *β*: 0.01; *p* = 0.01; 95% CI: [0.002; 0.02]). No statistically significant association was found between CpG_LV_ and any cell type, age or sex in the *SLC6A4* model.

Accounting for blood cells proportion did not affect the conclusions about the associations between *SLC6A4* or *TPH2* methylation and 5-HTT_LV_ or 5-HT_4LV_ in the healthy cohort nor the cohort of MDD patients (Table 2), as all *p*-values were greater or equal to 0.08 (Table 3).

**Table 3:** Association between a latent variable including blood cell proportions (CpG_LV+cells_) and a latent variable including serotonin transporter (5-HTT) or serotonin 4 receptor (5-HT_4_) binding

Brain 5-HT proxy	Cohort	Gene	Estimate	*P*-value	95% CI
5-HTT	HC	*SLC6A4*	− 0.01	0.95	[− 0.28; 0.26]
		*TPH2*	− 0.01	0.95	[− 0.28; 0.26]
5-HT_4_	HC	*SLC6A4*	− 0.22	0.08	[− 0.48; 0.03]
		*TPH2*	− 0.20	0.11	[− 0.45; 0.04]
	MDD	*SLC6A4*	0.08	0.56	[− 0.20; 0.37]
		*TPH2*	0.20	0.10	[− 0.04; 0.44]

*5-HT*: serotonin; *5-HTT*: serotonin transporter; *5-HT*_*4*_: serotonin 4 receptor; *HC*: healthy controls; *MDD*: patients with depression; *SLC6A4*: serotonin transporter gene; *TPH2*: tryptophan hydroxylase 2 gene

All models evaluating the association between *SLC6A4*/*TPH2* methylation and measures of early stress, anxiety or depressive symptoms and including cells proportions showed a significant association between lymphocytes proportions and CpG_LV_ (Table S8). Contrarily, granulocyte precursors were marginally statistically significantly associated only with *SLC6A4* CpG_LV_ in the model including healthy participants (Table S8). The other cell types considered showed no association. Age was statistically significantly associated with *TPH2* CpG_LV_ but not with *SLC6A4* CpG_LV_. Before adjusting for multiple comparisons, *TPH2* CpG_LV_ was associated with sex in the healthy participants but not in the MDD participants, with higher *TPH2* CpG_LV_ values in males compared to females (Table S8).

Associations between CpG_LV+cells_ and measures of environmental stress or mood or anxiety symptoms are depicted in Table S9 and showed no statistically significant association.

## Discussion

In this study we found no statistically significant associations between peripheral DNA methylation of two key regulatory genes of serotonin neurotransmission (*SLC6A4* and *TPH2)* and brain levels of 5-HTT and 5-HT_4_ in a cohort of healthy participants or 5-HT_4_ in a cohort of unmedicated patients with MDD.

Previous evidence supports an association between the CpG sites observed in our study and psychopathological features [7, 16, 48]. However, little is known about how peripheral DNA methylation of serotonin genes maps onto the brain serotonergic architecture. Only one study reported that increased *SLC6A4* methylation was associated with reduced in-vivo TPH2 brain levels in a cohort of adult males that experienced childhood aggression [27]. Nonetheless, the study was based on a relatively small cohort (*N* = 25) that experienced high childhood aggression while our study, although based on a notably larger cohort, includes participants that did not experience extreme childhood traumas.

The lack of an association observed in our study should be considered also in light of the intricate nature of gene regulation. First, DNA methylation levels can differ across tissues. Previous epigenome-wide association studies reported that methylation of some CpG sites correlate between peripheral blood cells and entorhinal cortex, cerebellum, superior temporal gyrus and prefrontal cortex in postmortem brains of elderly adults [29, 61]. To evaluate a correspondence of DNA methylation between the two tissues, we consulted the online database created by Hannon et al. (2015) and found that only *SLC6A4* CpG4 methylation levels correlate with methylation in entorhinal cortex (p_UNC_ = 0.02) and superior temporal gyrus (p_UNC_ = 0.04). *SLC6A4* CpG1 and *TPH2* CpG2 did not show any correlation, while information on the other CpG sites included in our study or other brain regions was not available in their database, so we cannot exclude a correspondence between the two tissues at other sites.

Second, even in the case DNA methylation was consistent across tissues, different transcription factors might interact differently with similar DNA methylation patterns in different tissues [62]. Thus, assuming similar DNA methylation levels between the two tissues, it is not known whether *SLC6A4* or *TPH2* expression would be affected in the brain in the same way that it is known to be affected in peripheral blood [13, 26, 27, 63]. Third, gene expression does not always directly correspond to protein levels as post-transcriptional and post-translational modifications can affect protein levels and function, and this notion seems to be true for both genes [55, 64–67]. This might also help explain why we did not observe any association between 5-HTTLPR or *TPH2* rs4570625, which are polymorphisms known to affect *SLC6A4* and *TPH2* expression, and 5-HTT or 5-HT_4_ levels, which is in line with former studies [50, 68].

Nonetheless, most of this evidence is based on studies in adult individuals and we cannot rule out an effect of genetic variation or DNA methylation within serotonergic genes on early brain development, which is critically driven by serotonin transmission [69, 70]. Indeed, while brain 5-HTT and 5-HT_4_ levels may vary substantially throughout the lifespan [35] and in response to environmental changes [71–74], DNA methylation remains stable at about half of the total CpG sites after the first years of life [75, 76]. Using the online database provided by Mulder et al. [76], we observed no change in DNA methylation at *SLC6A4* CpG1 or CpG4 or *TPH2* CpG2 over the first 17 years of life of healthy individuals, although information on the other CpG sites relevant to our study was not provided. Longitudinal study designs with methylation sampling and PET imaging would allow to better understand if this was the case.

Notably, the lack of association between peripheral DNA methylation and adult brain levels of serotonergic markers does not necessarily imply that *SLC6A4* or *TPH2* methylation cannot be used as an informative biomarker for mental health. Instead, it might reflect peripheral alterations, e.g. of the immune system which can be critical for mental health. Altered immune function has been described in individuals who have experienced early life stress [77] and stress-related disorders, including depression [78]. Likewise, previous findings relating *SLC6A4* and *TPH2* methylation to measures of early life stress [7, 79] or depressive symptoms [7] might reflect alterations in peripheral immune function rather than in the brain serotonergic transmission.

In this regard, our sensitivity analyses revealed a borderline significant association between *SLC6A4* CpG2 methylation at these genes and the parental bonding inventory (PBI) overprotection subscale, i.e. a proxy for suboptimal early social environment. However, this association was no longer statistically significant after including cell proportions in the model. Nonetheless, it is relevant to note that information of blood cells counts was available only for a subset of the total participants used for the sensitivity analyses, so such changes may be due to lower statistical power instead of the removal of unwanted variance. Thus, we suggest that this finding should be interpreted only if replicated in other cohorts.

Our study is the largest (*N* = 297) to date investigating the association between *SLC6A4*/*TPH2* DNA methylation and early life stress in a healthy cohort. In line with our study, the second largest study based on a cohort of healthy participants (*N* = 133) [41] reported no association between *SLC6A4* methylation and measures of early life stress. Our measurements for early life stress were PBI and SLE, which are based on retrospective self-reports and may not be as sensitive as other measurements in capturing early life stress. However, Wankerl et al. [41] did not find an association with early life stress, although both information of early-life stress reported by the participants’ mothers and self-reported were used. Thus, we can speculate that alterations in DNA methylation levels might only become detectable in case of more extreme (early) environmental stressors or in pathological conditions. Indeed, most previous studies linking peripheral *SLC6A4* or *TPH2* methylation to stress-related phenotypes were based on patients with mood disorders [7, 16] or individuals who were exposed to intense environmental stress [15, 27]. Our MDD cohort is smaller (*N* = 90) than some of those previously investigated (*N* > 100) [7, 16], so the results of our sensitivity analyses might be ascribable to a lack of power. Alternatively, the psychometric measurements used in our study might not be as sensitive at capturing early life stress as those used in other studies.

Importantly, the relation between *SLC6A4* and *TPH2* methylation and early life stress or brain levels of 5-HT_4_ or 5-HTT might also be affected by other environmental factors that were not considered in our study. For example, smoking [80], alcohol consumption [77, 81] or exposure to air pollutants [82] are known to affect gene expression through epigenetic modifications such as DNA methylation. Thus, we cannot exclude that future study designs including extreme exposure groups may inform on the potential effects of such environmental factors on a link between DNA methylation and serotonergic brain architecture.

In line with previous studies, we found that 5-HT_4_ binding was higher in men compared to women in healthy participants cohort [59] but not in the MDD cohort [83]. Previous studies investigating *SLC6A4* and *TPH2* methylation suggests that DNA methylation levels might be affected by sex [79, 84]. However, in our dataset we did not observe any conclusive effects of sex on neither gene, except for a trend in *TPH2* CpG_LV_ in the healthy participants. Notably, we observed it only before correcting for multiple comparisons, which we therefore interpret with caution. We did not observe the same effect on the MDD cohort.

It is important to mention some strengths of the present study compared with previous literature. First, it is based on the currently largest dataset in the world for brain molecular imaging for 5-HTT and 5-HT_4_. This also allows us to validate previous findings based on the same cohort such as in the case of *BDNF* rs6265 and 5-HT_4_ [58], which was initially found in a subset (*N *= 68) of the participants included in this cohort but could not be replicated in the more recent and larger current cohort (*N *= 112). Second, it includes both healthy participants and MDD patients, allowing us to investigate potential associations unique to healthy or pathological states; third, in our analyses we included blood cells proportions, which has rarely been done in former studies evaluating methylation at *SLC6A4* or *TPH2* and can be the main driver of methylation variability across individuals [39, 85].

However, this study also comes with several limitations. First, we examined four and six CpG sites for *SLC6A4* and *TPH2* respectively, which is only a small fraction of the total CpG sites in these genes and even smaller of those across the genome. An epigenome-wide exploration would be more informative, although a much larger sample size would be needed to capture potential peripheral epigenetic signatures associated with brain serotonergic transmission. For this reason, we chose a candidate epigenetic marker strategy for this study. Second, we considered only 5-HTT and 5-HT_4_ as proxies for serotonergic neurotransmission. Although this was based on previous evidence, there are many more contributors to serotonergic neurotransmission. In addition, in the MDD cohort we were only able to explore the association between DNA methylation and 5-HT_4_ levels since we did not have data on 5-HTT brain binding from this group. Alterations in brain 5-HTT levels have been reported in MDD patients [86] and future studies are needed to explore the relation between peripheral *SLC6A4* methylation and brain 5-HTT levels in MDD. Nonetheless, preclinical and clinical studies show that 5-HT_4_ levels vary in response to serotonin levels [32, 87, 88] and in-vivo 5-HT_4_ levels deviate between healthy participants and MDD patients [36], pointing to the relevance of this target as a proxy for serotonin transmission in the context of MDD.

Third, although we included cell proportions in our sensitivity analyses, we could not take into account all blood cells subtypes (e.g. lymphocyte subtypes such as CD4+ or CD8+) but only the broader classes or cell types (monocytes, lymphocytes, neutrophils and granulocytes precursors) that are commonly evaluated in clinical routine. Thus, although we have accounted for some of the variance deriving from blood cell composition, we cannot assume that our analyses have accounted for all the variance.

Fourth, we could not account for the timing of possible traumas experienced by our participants. Cumulative evidence shows that the timing at which environmental stress was experienced can differentially affect DNA methylation [89, 90] as well as the vulnerability to developing psychiatric disorders [91, 92]. Future studies carried out on naturalistic cohorts should consider collecting data on the timing but also on the type of stress experienced in early life, to better capture the individual exposome. Finally, DNA methylation is tightly associated with genetic variation [93]. Several studies reporting associations with early life stress or mood disorder symptoms are based on populations of Asian ancestry, whereas our study only included participants of European ancestry. Hence, although we took into account two genetic variants that might influence the expression of *SLC6A4* or *TPH2*, our findings may only be generalizable to populations of European ancestry. In addition, we used the online mQTL database browser (http://www.mqtldb.org/) to investigate potential genetic influences on the methylation loci considered in our study. We did not find any variant associated with any of our CpG sites of interest. However, as only three out of the ten loci were available on their dataset, we cannot exclude that other genetic variants might affect *SLC6A4* or *TPH2* methylation. Future studies considering more CpG sites and genetic variants as well as including participants from different ancestries would help clarify this limitation.

## Conclusions

To conclude, our findings do not support an association between *SLC6A4* or *TPH2* methylation and 5-HTT or 5-HT_4_ brain levels or measures of early life stress, anxiety or depressive symptoms. We suggest that caution should be used when interpreting findings on peripheral DNA methylation in relation to the adult serotonergic brain architecture and to measures of early life stress or mood disorders symptoms. However, our findings do not rule out a role of peripheral DNA methylation in serotonergic neurotransmission and (mal)adaptation to environmental stress, which should be further elucidated by future studies considering more CpG sites and related genetic variants, larger sample sizes, more sensitive measures of early environmental stress, blood cell composition and longitudinal cohorts.

### Supplementary Information

Table S1. Demographics of the participants included in the secondary analyses (XLSX 12 kb)

Table S2. Genomic locations of the *SLC6A4* and *TPH2* CpG sites included in this study (XLSX 10 kb)

Table S3. Primers used for pyrosequencing *SLC6A4* and *TPH2* (XLSX 9 kb)

Table S4. Effect sizes and respective 95% confidence intervals of models including *SLC6A4* data (XLSX 11 kb)

Table S5. Effect sizes and respective 95% confidence intervals of models including *TPH2* data (XLSX 12 kb)

Table S6. Associations between *SLC6A4* methylation and environmental stress in healthy controls (HC) and mood and anxiety disorder symptoms in patients with depression (MDD) (XLSX 11 kb)

Table S7. Associations between *TPH2* methylation and environmental stress in healthy controls (HC) and mood and anxiety disorder symptoms in patients with depression (MDD) (XLSX 12 kb)

Table S8. Results of associations between CpG_LV+cells_ and cell proportions, sex, age and genotype (XLSX 10 kb)

Table S9. Results of associations between CpG_LV+cells_ and measures of environmental stress or mood or anxiety symptoms (XLSX 10 kb)

## Data Availability

The R codes used for statistical analyses can be made available upon request to the corresponding author (vibe.frokjaer@nru.dk). Data can be made available upon reasonable request via this form (https://cimbi.dk/index.php/documents/category/3-cimbi-database) and with an appropriate data sharing agreement.

## Abbreviations

*SLC6A4*: Serotonin transporter gene
*TPH2*: Tryptophan hydroxylase 2 gene
5-HTT: Serotonin transporter
5-HT_4_: Serotonin 4 receptor
MDD: Major depressive disorder
5-HTTLPR: Serotonin-transporter-linked promoter region polymorphism
CpG: Cytosine–Guanine dinucleotide
HAMD17: Hamilton depression rating scale 17-item
HAMD6: Hamilton depression rating scale 6-item
MRTM/MRTM2: Multilinear reference tissue model
LVM: Latent variable model
5-HTT_LV_: Latent variable based on 5-HTT binding values
5-HT_4LV_: Latent variable based on 5-HT_4_ binding values
ROI: Region of interest
SLE: Stressful life events
PBI: Parental bonding inventory
BDI: Beck’s depression index
CATS: Childhood abuse trauma scale
GAD10: Generalized anxiety disorder 10-item
PSS: Perceived stress scale
CpG_LV_: Latent variable based on CpG values
CpG_LV+cells_: CpG_LV_ adjusted for cell proportions
HC: Healthy control
